# OPUS-BFactor: Predicting Protein B-Factor with Sequence and Structure Information

**DOI:** 10.3390/molecules30122570

**Published:** 2025-06-12

**Authors:** Yulu Yang, Ying Lv, Zhenwei Luo, Qinghua Wang, Gang Xu, Jianpeng Ma

**Affiliations:** 1 Multiscale Research Institute of Complex Systems, Fudan University, Shanghai 200433, China; 19110700090@fudan.edu.cn (Y.Y.); luozhenwei@pjlab.org.cn (Z.L.); 2 Shanghai AI Laboratory, Shanghai 200030, China; lvying@pjlab.org.cn; 3 Center for Biomolecular Innovation, Harcam Biomedicines, Shanghai 200131, China; qinghuawang9@gmail.com

**Keywords:** protein B-factor, protein language model, protein flexibility

## Abstract

Protein B-factor, also known as the Debye–Waller temperature factor or atomic displacement parameter, measures the thermal fluctuation of an atom around its average position. It serves as a crucial indicator of protein flexibility and dynamics. However, accurately predicting the B-factor of Cα atoms remains challenging. In this work, we introduce OPUS-BFactor, a tool for predicting the normalized protein B-factor. OPUS-BFactor employs a transformer-based module to integrate sequence-level and pair-level features, encompassing structural attributes derived from the protein’s 3D structure and evolutionary profiles obtained from the protein language model ESM-2. Specifically, OPUS-BFactor treats pair features as a bias term, incorporating them into the attention matrix derived from the sequence-level features of each residue pair, thereby effectively merging pair features with sequence features. OPUS-BFactor operates in two modes, enabling predictions based solely on either the protein sequence or the 3D structure of the target protein. Evaluation on three test sets, including recently released targets from CAMEO and CASP15, demonstrated that OPUS-BFactor significantly outperformed other B-factor prediction methods. Therefore, OPUS-BFactor is a valuable tool for predicting protein properties related to the B-factor, such as flexibility, thermal stability, and regional activity.

## 1. Introduction

Protein B-factor, also known as the Debye–Waller factor, atomic displacement parameter, or temperature factor, measures the mean squared displacement or uncertainty of atomic positions [1,2,3]. Numerous studies have shown that protein B-factor is valuable in various areas, such as predicting protein flexibility [4,5], evaluating thermal stability [6], analyzing active and disordered regions [7,8], and studying protein dynamics [9]. Since protein fluctuation provides a crucial link between structure and function [2,10], accurately predicting protein B-factor is essential for understanding the characteristics of target proteins. Recently, several studies have highlighted the limitations associated with the utilization of B-factors, underscoring the crucial significance of rescaling B-factors in protein crystal structure analyses [11,12,13].

Over the past several decades, numerous methods have been proposed for predicting protein B-factor [2,14,15,16,17,18,19]. Some studies have introduced normal mode analysis (NMA) into this field [10,20,21,22]. In NMA, the Hessian of the harmonic potential is employed to describe the atomic thermal fluctuations; therefore, the B-factors of proteins are correlated with the eigenvalues of the Hessian. Meanwhile, the Gaussian network model (GNM) and the anisotropic network model (ANM) are two elastic network models that have been widely used to study protein fluctuation dynamics [23,24,25].

In recent years, several machine learning-based models have been proposed for predicting protein B-factors [2,14,15,26,27]. Some of these models utilize support vector regression (SVR) [14,15,26], and some of them employ graph models, such as multiscale weighted colored graphs [27]. With the development of deep learning techniques [28], several new methods based on deep learning frameworks have emerged [16,18,29]. Most of these methods [16,18] adopt the bidirectional long short-term memory (BiLSTM) network [30]. Additionally, the method proposed by Sarparast et al. [29] utilizes a graph-based network to capture structural features from protein 3D structures, significantly enhancing the accuracy of B-factor prediction.

In this study, we introduce a deep learning-based model named OPUS-BFactor for predicting protein B-factors (specifically for C_α_ atoms). OPUS-BFactor operates in two modes. In the first mode, it uses sequence information as input, enabling predictions based solely on protein sequence. Previous sequence-based methods typically rely on one-hot encoding (representing residues as 20-dimensional binary vectors where each vector corresponds to a specific residue type) [16] or evolutionary features such as PSSM profiles (position-specific scoring matrix) and HMM profiles (hidden Markov model) [18]. Recently, numerous protein language models have been developed, significantly improving the quality of the extracted evolutionary features [31,32,33,34]. Among them, ESM-2 [35] stands out as the most widely used, with applications spanning numerous domains [36,37,38]. Consequently, in this study, OPUS-BFactor utilizes evolutionary features derived from the protein language model ESM-2. The results show that using ESM-2 features as inputs significantly improves B-factor prediction accuracy compared with those using one-hot encoding and PSSM features. In the second mode, OPUS-BFactor utilizes structural information, achieving better results than the sequence-based mode. For clarity, we refer to the results of OPUS-BFactor based on sequence information as OPUS-BFactor-seq (first mode) and the results based on structural information as OPUS-BFactor-struct (second mode).

We assessed the performance of OPUS-BFactor using three test sets: CAMEO65, CASP15, and CAMEO82. The results indicated that OPUS-BFactor-struct significantly outperformed other methods. Specifically, on the most recently released CAMEO82 test set, the average Pearson correlation coefficient (PCC) for B-factor from C_α_ atoms was 0.67 for OPUS-BFactor-struct and 0.58 for OPUS-BFactor-seq, compared with 0.41 for the most recent method proposed by Pandey et al. [16].

Although many methods have been proposed for protein B-factor prediction, they usually use different training and test sets, making fair comparisons difficult. Additionally, the code for many methods is not publicly available, complicating their use by other researchers. Therefore, we will make our training and test sets, as well as our code, available to all researchers. We hope that OPUS-BFactor will serve as a fair baseline method in protein B-factor prediction. Additionally, the formatted datasets may become a useful benchmark to facilitate the development of protein language models, given that the performance of sequence-based B-factor prediction models still lags behind that of structure-based models.

## 2. Results

### 2.1. Performance of Different B-Factor Prediction Methods

We evaluated the performance of OPUS-BFactor against a normal mode analysis (NMA)-based method, ProDy [39], and a deep learning-based method developed by Pandey et al. [16] across three test sets (CAMEO65, CASP15, and CAMEO82). As shown in Table 1, OPUS-BFactor consistently surpassed other methods in terms of average PCC on all three test sets. Additionally, the structure-based mode of OPUS-BFactor (OPUS-BFactor-struct) delivered better results than its sequence-based version (OPUS-BFactor-seq), indicating that structural information was crucial for accurate protein B-factor prediction.

**Figure 1:**
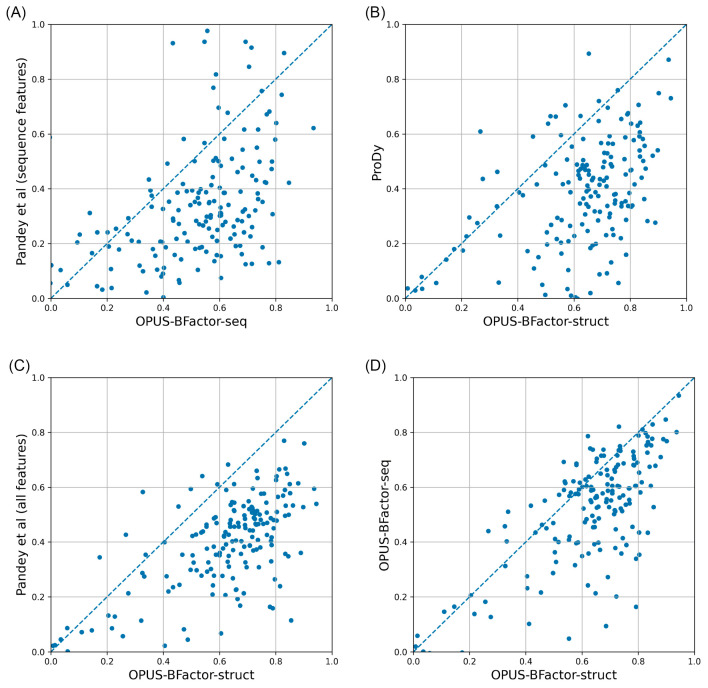
The head-to-head comparison of the average PCC achieved by different methods. (**A**) Comparison between two sequence-based methods: OPUS-BFactor-seq and Pandey et al. (sequence features). (**B**) Comparison between two structure-based methods: OPUS-BFactor-struct and ProDy. (**C**) Comparison between two structure-based methods: OPUS-BFactor-struct and Pandey et al. (all features). (**D**) Comparison between two modes of OPUS-BFactor: OPUS-BFactor-struct and OPUS-BFactor-seq.

We combined the targets from all three test sets, resulting in a complete dataset comprising 181 targets, and then conducted a head-to-head comparison by analyzing the average Pearson correlation coefficient (PCC) for each target across different methods. As shown in Figure 1, the structure-based mode of OPUS-BFactor, specifically OPUS-BFactor-struct, demonstrated superior performance in most cases, outperforming the other evaluated methods.

**Figure 2:**
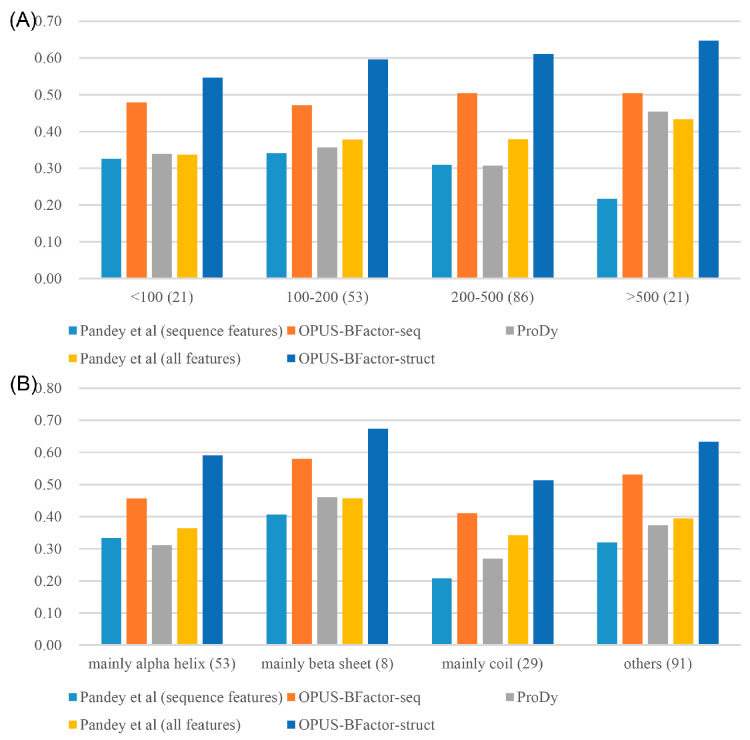
The average PCC achieved by different methods [16,39] on the targets categorized by their lengths and subfamilies. (**A**) The average PCC of different methods on the targets with varying lengths. (**B**) The average PCC of different methods on the targets belonging to distinct subfamilies. The subfamilies were primarily defined based on the secondary structure of the targets, with the categories of mainly alpha helix, beta sheet, and coil representing targets where more than 50% of the residues belonged to the respective secondary structure types. The numbers enclosed in parentheses denote the total count of targets within each of the subgroups.

Furthermore, we conducted a comparative analysis of the average PCC among various methods, stratified by the lengths and subfamilies of all 181 targets. As depicted in Figure 2, OPUS-BFactor-struct and OPUS-BFactor-seq consistently outperformed other methods across various target lengths and subfamilies. However, it was noteworthy that the PCCs of all the methods exhibited a decline when evaluated on targets predominantly characterized by coil structure.

**Figure 3:**
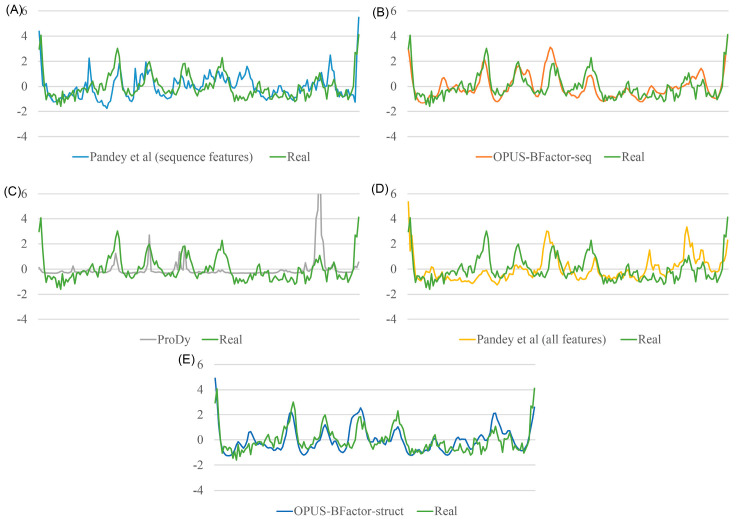
Protein B-factor prediction results of different methods [16,39] against the real normalized protein B-factor values for the target 2023-05-06_00000171_1. (**A**) Comparison between real values and predictions from Pandey et al. (sequence features). (**B**) Comparison between real values and predictions from OPUS-BFactor-seq. (**C**) Comparison between real values and predictions from ProDy. (**D**) Comparison between real values and predictions from Pandey et al. (all features). (**E**) Comparison between real values and predictions from OPUS-BFactor-struct.

In Figure 3 andFigure 4, we present some prediction results obtained from each method. These results showed that OPUS-BFactor was capable of achieving satisfactory results for most of the residues on these targets.

### 2.2. Correlation Between Protein B-Factors and the pLDDT Values from Structure Prediction Methods

In recent years, numerous highly effective protein structure prediction methods have been proposed, significantly advancing the field of computational biology. To investigate the correlation between the protein B-factor and the predicted local distance difference test (pLDDT) values derived from these prediction methods, we initially employed the state-of-the-art ESMFold [35] model to predict the structures of targets within our three test sets. Subsequently, we calculated the average PCC between the real B-factor values and the predicted pLDDT scores. Given that the pLDDT score served as an indicator of the prediction uncertainty, where smaller values indicated reduced confidence in the corresponding region, we chose to employ negative pLDDT values in our correlation analysis to assess the feasibility of utilizing pLDDT scores as a proxy for protein B-factors. Note that while pLDDT values were always positive, they exhibited an inverse relationship with B-factors regarding structural flexibility: lower pLDDT values indicated higher flexibility, whereas higher B-factors indicated higher flexibility. To ensure consistent interpretation, we used negative pLDDT values in this study.

As shown in Table 2, the average PCCs between the real B-factors and pLDDT values were notably lower compared with those achieved by our sequence-based approach, OPUS-BFactor-seq. This indicated a relatively weak correlation between protein B-factors and the pLDDT values, necessitating the development of tailored approaches for predicting protein B-factors.

Meanwhile, Table 3 presents a disaggregated analysis of the average PCCs for both approaches, classified by the structural prediction accuracy of ESMFold for the entire set of 181 targets. The results demonstrated that as the structural prediction difficulty of the targets increased, there was a corresponding decrease in the accuracy of B-factor prediction when relying solely on sequence information. When using real PDB structures, OPUS-BFactor-struct exhibited only a negligible decrease in PCC between targets with TM score >0.9 and those with TM scores between 0.8 and 0.9. This demonstrated that OPUS-BFactor-struct maintained reasonably good performance for easily predicted targets. Additionally, compared with results derived from real PDB structures, those based on ESMFold exhibited inferior performance. Notably, this performance gap widened as the quality of the predicted structure decreased.

Furthermore, we used AlphaFold2 [40] to predict the structures of 44 targets in the CASP15 test set and calculated the PCC between the real B-factors and their corresponding pLDDT values. The results showed that the average PCC between the real B-factors and the pLDDT values from AlphaFold2 on the CASP15 test set was 0.23, which was even lower than the PCC achieved by ESMFold (0.24 in Table 2). Consequently, this finding also indicated a relatively weak correlation between B-factors and pLDDT values, which was consistent with earlier observations reported by Carugo et al. [41].

### 2.3. Evaluation of Different Evolutionary Profiles

We evaluate the performance of sequence-based B-factor prediction models (OPUS-BFactor-seq) using different evolutionary profiles on the CAMEO82 test set. As shown in Figure 5, the model using ESM-2 features as inputs significantly improved prediction accuracy compared with models using (1) one-hot encoding (represented as a 20-dimensional binary vector indicating residue identity), (2) HMM profiles (generated using hh-suite), or (3) PSSM features (derived from BLAST2.14 alignments). This indicated that the performance of protein B-factor prediction could be enhanced by the utilization of more advanced evolutionary features.

### 2.4. Case Study

In this study, we utilized OPUS-BFactor-seq to predict the B-factors for T4 lysozyme and the tumor suppressor p53 based on their sequences exclusively. As shown in Figure 6A, we highlight two regions (regions A and B) in the prediction with relatively high values on the T4 lysozyme. The studies from other researchers show that region A (D20-G23) corresponds to the active site of T4 lysozyme [42], and region B (K35-L39) is a relatively flexible region as some studies indicate that an insertion or duplication of short peptide fragments in this area may cause a secondary structural transition (from helix to strand) [43]. Furthermore, in Figure 6B, we observe a region with relatively high B-factor values in tumor suppressor p53 (region C), which is related to its DNA-binding site [44]. Our results suggest that OPUS-BFactor could effectively predict B-factor-related properties like flexibility, thermal stability, and functional activity.

## 3. Method

### 3.1. Framework of OPUS-BFactor

OPUS-BFactor adopts the RotaFormer module from OPUS-Rota5 [45] as its backbone architecture, with some modifications. As shown in Figure 7, the 1D and 2D features are derived from protein structural information, while the ESM-2 features are obtained from the protein language model ESM-2 [35], which relies solely on protein sequence. Specifically, the 1D protein features include two one-hot encoded features for the 3-state and 8-state secondary structures, seven physicochemical properties [46,47], 19 PSP features representing 19 rigid-body blocks within residues [47,48,49], and six backbone torsion angle features (sine and cosine values for ϕ, ψ, and ω). The 2D features describe residue–residue backbone contact information [50,51], including C_β_–C_β_ distance distributions and orientational distributions of three dihedrals (ω, θ_ab_, and θ_ba_) and two angles (φ_ab_ and φ_ba_) between residues a and b. Here, ω represents the dihedral of Cαa–Cβa–Cβb–Cαb, θab represents the dihedral of Na–Cαa–Cβa–Cβb, and φab represents the angle of Cαa–Cβa–Cβb. Distances of C_β_–C_β_ span from 2 to 20 Å, segmented into 36 bins at 0.5 Å intervals, with an additional bin for distances exceeding 20 Å. The φ angle ranges from 0 to 180°, divided into 18 bins at 10° intervals, with an extra bin for non-contact scenarios. Both ω and θ range from −180 to 180°, segmented into 36 bins at 10° intervals, with an extra bin for non-contact scenarios. The ESM-2 features include a 1280-dimensional feature for each residue, containing their evolutionary information.

In OPUS-BFactor, the embedding module transforms the 1D features into sequence and pair features. The 2D features are processed through a 2D convolution layer and then added to the pair features, while the ESM-2 features are passed through a dense layer and added to the sequence features. Next, the RotaFormer module [45] is used to integrate the sequence and pair features. OPUS-BFactor employs 24 RotaFormers for feature extraction. After that, four BiLSTM layers [30] are used to further aggregate the sequence features and output the predicted B-factor value of C_α_ atoms. Following Pandey et al. [16], since the normalized B-factor has been shown to be more robust against experimental noise [1], we used the normalized B-factor in this study.

During training, we used the mean absolute error (MAE) loss between the predicted and actual normalized B-factor. We employed the Glorot uniform initializer and the Adam optimizer [52]. However, one notable limitation of OPUS-BFactor is that despite the sophistication of its transform-based model, it requires substantial computational resources, with each epoch taking nearly 15 h to complete. To address this, we initiated the training process with a learning rate of 1 × 10^3^ and halved it every two epochs. Additionally, we randomly selected 90% of the training data for model training. The training was conducted for a total of six epochs. Following this, the remaining 10% of the data was utilized as a validation set to select the optimal model. To mitigate overfitting and enhance generalization, we employed early stopping with a patience of 4 evaluations, selecting the best model checkpoint based on peak validation performance. Validation was performed every 1500 training steps. OPUS-BFactor was developed using TensorFlow v2.4 [53] and trained on four NVIDIA Tesla V100 GPUs.

### 3.2. Datasets

In OPUS-BFactor, we used the same training dataset as trRosetta [50]. Additionally, we removed proteins where all residues shared identical B-factors. To evaluate the performance across various methods, we utilized three recently released test sets. The first, CAMEO65, was collected by Xu et al. [54] and contained 65 challenging targets released between May 2021 and October 2021 from the CAMEO website [55]. After filtering, 62 targets remained. The second test set, CASP15, included 44 targets available from the CASP website (http://predictioncenter.org (accessed on 1 January 2020)). The third, CAMEO82, was collected by Xu et al. [39] and contained 82 targets released between May 2023 and August 2023 from the CAMEO website, with 75 targets remaining after filtering. In this study, we used the normalized B-factor (B) for each C_α_ atom as the corresponding labels, calculated using the formula B = (B − μ)/σ, where μ and σ are the mean and standard deviation of the unnormalized B-factor value (B) within the target protein.

### 3.3. Performance Metrics

To evaluate the accuracy of each method, we used the average Pearson correlation coefficient (PCC) for each test set as our metric.

### 3.4. Data and Software Availability

The code and pre-trained models of OPUS-BFactor, as well as the datasets used in the study, can be downloaded from http://github.com/OPUS-MaLab/opus_bfactor (accessed on 1 January 2020).

## 4. Concluding Discussion

**Figure 4:**
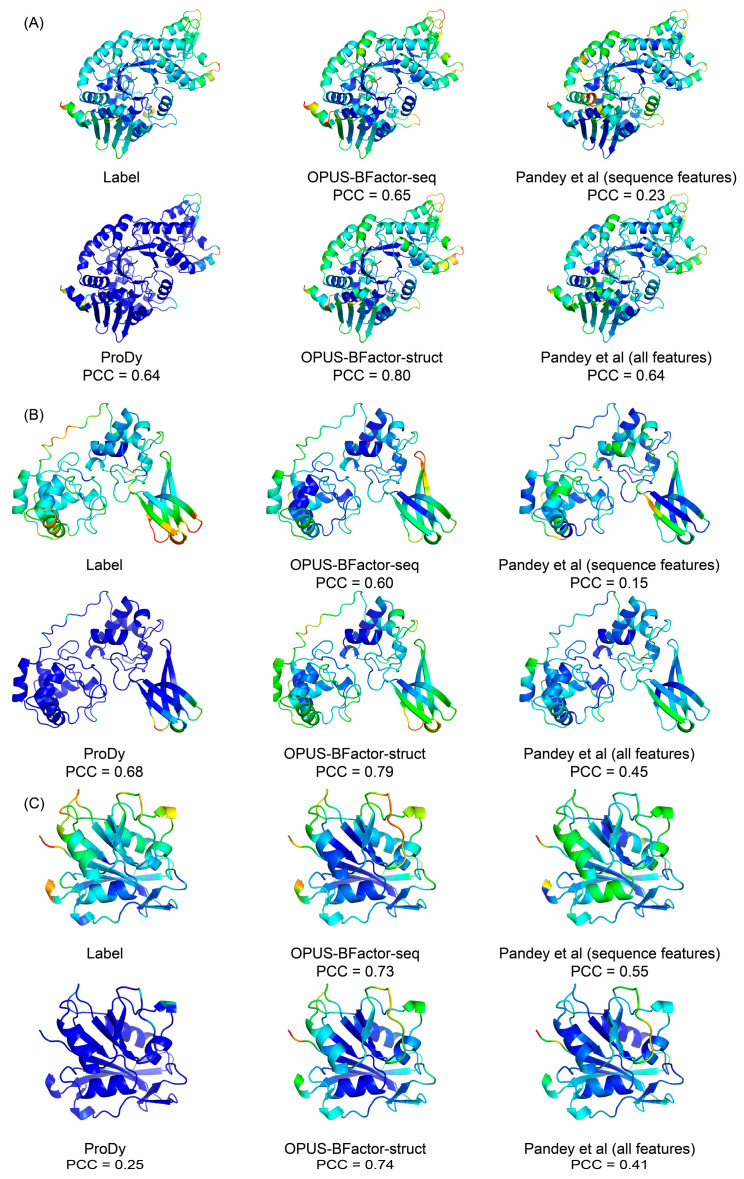
Protein B-factor prediction results of different methods. Results are colored in a spectrum (from blue to red) according to the B-factor of each C_α_ atom using PyMOL software-v2.4. (**A**) Prediction results on 2023-05-06_00000066_1. (**B**) Prediction results on 2023-05-13_00000063_1. (**C**) Prediction results on 2023-05-06_00000171_1.

In this study, we propose a protein B-factor prediction method called OPUS-BFactor, which operates in two modes: the first one (OPUS-BFactor-seq) uses sequence information exclusively, allowing predictions based solely on protein sequence, and the second one (OPUS-BFactor-struct) utilizes structural information, requiring the coordinates of backbone atoms in the target protein. The results (Table 1, Figure 1, Figure 2, Figure 3 and Figure 4) on three recently released test sets showed that our method significantly outperformed other B-factor prediction methods. Meanwhile, the results highlight a performance gap between sequence-based and structure-based B-factor prediction models; the latter is significantly better than the former.

It should be noted that most of the previous methods employed the LSTM architecture as their neural network backbone. In contrast, OPUS-BFactor is based on a more sophisticated transformer-based network architecture (i.e., RotaFormer module in Figure 7), which is capable of integrating features between the sequence level and the pair level more effectively. Specifically, in OPUS-BFactor, sequence-level features are integrated with pair-level features through an outer product operation. Meanwhile, pair-level features serve as a bias term to integrate with the attention matrix derived from sequence-level features. Moreover, given that most previous methods relied on traditional evolutionary features such as PSSM or HMM profiles, the superior performance of OPUS-BFactor can also be largely attributed to its utilization of more powerful evolutionary features derived from ESM-2.

We also evaluated the correlation between real B-factors and predictions from OPUS-BFactor-seq, as well as the correlation between real B-factors and the pLDDT from ESMFold and AlphaFold2; all of them relied on sequence information exclusively. The results (Table 2 and Table 3) showed that OPUS-BFactor-seq delivered better results. In this case, B-factor prediction methods, such as OPUS-BFactor, can be utilized to provide additional information regarding protein flexibility for the structure prediction methods such as ESMFold and AlphaFold2.

Additionally, the results on T4 lysozyme (Figure 6A) and the tumor suppressor p53 (Figure 6B) indicated that the regions with relatively high values of B-factors predicted by OPUS-BFactor-seq corresponded with the active/binding sites and flexible regions of the target. Therefore, OPUS-BFactor-seq may serve as a useful tool for predicting protein properties related to the B-factor, such as flexibility, thermal stability, and regional activity.

**Figure 5:**
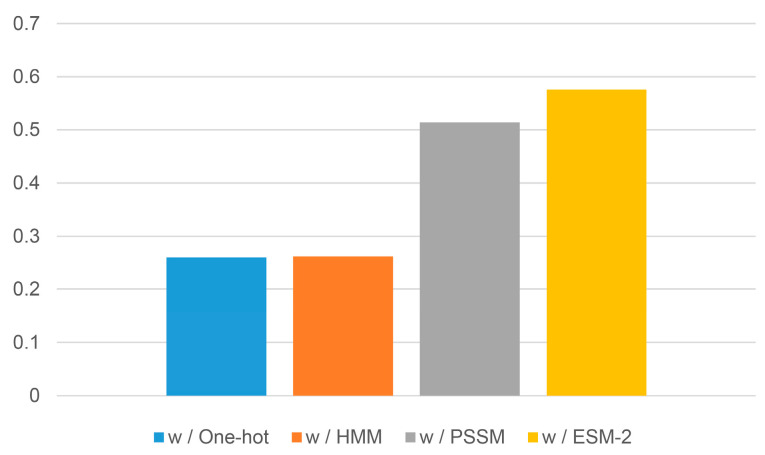
The average Pearson correlation coefficient (PCC) of OPUS-BFactor-seq using different evolutionary features as inputs on CAMEO82.

**Figure 6:**
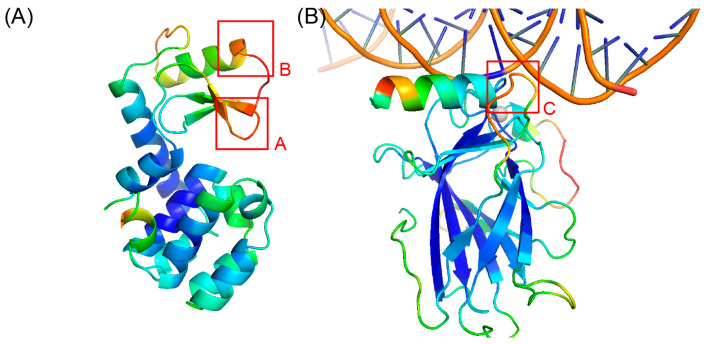
OPUS-BFactor-seq prediction results using the sequence information exclusively. (**A**) Results for the T4 lysozyme. (**B**) Results for the tumor suppressor p53. The results are colored in a spectrum (from blue to red) according to the B-factor of each C_α_ atom using PyMOL software-v2.4. The B-factors of the two residues located at both ends of the sequences were set to 0. In (**A**), region A includes residues between 20 and 23, and region B includes residues between 35 and 39. In (**B**), region C includes residues between 116 and 124.

**Figure 7:**
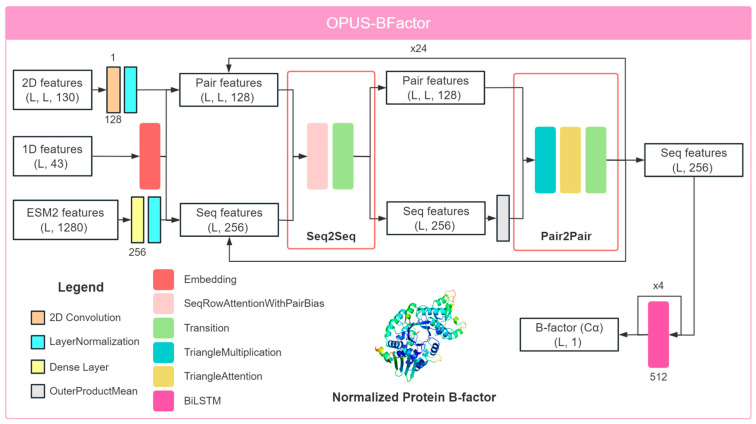
Overview of the OPUS-BFactor framework. OPUS-BFactor takes in three primary inputs: 1D protein sequence features, 2D residue–residue contact features, and 1D ESM-2 protein evolutionary features. In the first mode, OPUS-BFactor-seq, the structure-based features (first two) are set to zero, allowing predictions to be based solely on the protein sequence. In the second mode, OPUS-BFactor-struct, the ESM-2 features are set to zero. For a target protein, OPUS-BFactor predicts the normalized B-factor of C_α_ atoms for all residues.

Furthermore, the results (Figure 5) showed that the performance of protein B-factor prediction may benefit from more advanced evolutionary features. In this case, protein B-factor prediction could serve as a valuable benchmark task for assessing protein language models. To facilitate this, we will make our formatted training and test sets, along with our code, available to all researchers.

Although OPUS-BFactor achieved a relatively high correlation with the B-factors from the PDB file, it should be noted that further investigation is needed to implement stricter filtering. This is because B-factors can be influenced by various factors beyond conformational flexibility, such as static disorder, crystal packing effects, and experimental noise. Furthermore, OPUS-BFactor currently cannot reliably differentiate B-factor variations between closely related protein structures. This limitation suggests an important direction for future improvement, potentially through the integration of additional structural or evolutionary information into the model. Such refinements could improve predictive accuracy and expand its applications, such as assessing the quality of predicted protein structures.

## Figures and Tables

**Table 1:** The average (median) Pearson correlation coefficient (PCC) of different methods on each test set.

	CAMEO65	CASP15	CAMEO82
Sequence-based methods			
Pandey et al. [16]	0.37 (0.30)	0.20 (0.24)	0.33 (0.30)
OPUS-BFactor-seq	0.50 (0.56)	0.34 (0.41)	0.58 (0.60)
Structure-based methods			
ProDy [39]	0.31 (0.33)	0.25 (0.28)	0.43 (0.44)
Pandey et al. [16]	0.38 (0.42)	0.33 (0.35)	0.41 (0.43)
OPUS-BFactor-struct	0.61 (0.69)	0.48 (0.56)	0.67 (0.69)

**Table 2:** The average PCC between the real B-factors and the predicted B-factors generated by OPUS-BFactor-seq, as well as the average PCC between the real B-factors and the pLDDT values derived from the ESMFold predicted structures on each test set.

	CAMEO65	CASP15	CAMEO82
pLDDT (ESMFold)	0.28	0.24	0.38
OPUS-BFactor-seq	0.50	0.34	0.58

**Table 3:** The average PCC achieved by different methods on the targets categorized based on the structural prediction accuracy of ESMFold. The numbers enclosed in parentheses denote the total count of targets within each of subgroups.

	TM Score > 0.9 (89)	0.8 < TM Score < 0.9 (32)	Others (60)
pLDDT (ESMFold)	0.42	0.34	0.14
OPUS-BFactor-seq	0.61	0.48	0.33
OPUS-BFactor-struct (ESMFold)	0.63	0.55	0.34
OPUS-BFactor-struct (PDB)	0.67	0.64	0.48

## Data Availability

The code and pre-trained models of OPUS-BFactor, as well as the datasets used in the study, can be downloaded from http://github.com/OPUS-MaLab/opus_bfactor (accessed on 1 January 2025).

## Abbreviations

The following abbreviations are used in this manuscript:

pLDDT	predicted local distance difference test
